# Extra-platelet low-molecular-mass thiols mediate the inhibitory action of *S*-nitrosoalbumin on human platelet aggregation via *S*-transnitrosylation of the platelet surface

**DOI:** 10.1007/s00726-021-02950-8

**Published:** 2021-02-14

**Authors:** Dimitrios Tsikas

**Affiliations:** Institute of Toxicology, Core Unit Proteomics, Hannover Medical School, Carl-Neuberg-Strasse 1, 30625 Hannover, Germany

**Keywords:** Aggregation, *S*-Nitrosothiols, Platelets, PTM, Thiols

## Abstract

Nitrosylation of sulfhydryl (SH) groups of cysteine (Cys) moieties is an important post-translational modification (PTM), often on a par with phosphorylation. *S*-Nitrosoalbumin (ALB-Cys^34^SNO; SNALB) in plasma and *S*-nitrosohemoglobin (Hb-Cys^β93^SNO; HbSNO) in red blood cells are considered the most abundant high-molecular-mass pools of nitric oxide (NO) bioactivity in the human circulation. SNALB per se is not an NO donor. Yet, it acts as a vasodilator and an inhibitor of platelet aggregation. SNALB can be formed by nitrosation of the sole reduced Cys group of albumin (Cys^34^) by nitrosating species such as nitrous acid (HONO) and nitrous anhydride (N_2_O_3_), two unstable intermediates of NO autoxidation. SNALB can also be formed by the transfer (*S*-transnitrosylation) of the nitrosyl group (NO^+^) of a low-molecular-mass (LMM) *S*-nitrosothiol (RSNO) to ALB-Cys^34^SH. In the present study, the effects of LMM thiols on the inhibitory potential of ALB-Cys^34^SNO on human washed platelets were investigated. ALB-Cys^34^SNO was prepared by reacting *n*-butylnitrite with albumin after selective extraction from plasma of a healthy donor on HiTrapBlue Sepharose cartridges. ALB-Cys^34^SNO was used in platelet aggregation measurements after extended purification on HiTrapBlue Sepharose and enrichment by ultrafiltration (cutoff, 20 kDa). All tested LMM cysteinyl thiols (R-CysSH) including l-cysteine and L-homocysteine (at 10 µM) were found to mediate the collagen-induced (1 µg/mL) aggregation of human washed platelets by SNALB (range, 0–10 µM) by cGMP-dependent and cGMP-independent mechanisms. The LMM thiols themselves did not affect platelet aggregation. It is assumed that the underlying mechanism involves *S*-transnitrosylation of SH groups of the platelet surface by LMM RSNO formed through the reaction of SNALB with the thiols: ALB-Cys^34^SNO + R-CysSH ↔ ALB-Cys^34^SH + R-CysSNO. Such *S*-transnitrosylation reactions may be accompanied by release of NO finally resulting in cGMP-dependent and cGMP-independent mechanisms.

**Supplementary Information:**

The online version contains supplementary material available at 10.1007/s00726-021-02950-8.

## Introduction

Human serum albumin (HSA) or human plasma albumin (ALB) is one of the most abundant circulating proteins with numerous physiological functions and roles in health and various diseases including cardiovascular disease (Chien et al. 2017; Argues 2020). Albumin undergoes numerous post-translational modifications (PTM) which may be associated with clinical implications related and non-related to oxidative stress (Colombo et al. 2012; Watanabe et al. 2017). Most frequent and best investigated PTM on albumin include *N*-glycosylation (glycation) on lysine (*n* = 22) and asparagine (*n* = 2) residues, phosphorylation on serine (*n* = 6) and threonine (*n* = 3) residues, *N*^ε^-succinylation (*n* = 4) and *N*^ε^-methylation (*n* = 1) of lysine residues (Rondeau and Bourdon 2011; https://www.uniprot.org/uniprot/P02768#ptm_processing).

PTM in albumin also occur on the sole residue of reduced cysteine (Cys^34^) to form disulfides with cysteine, glutathione and other cysteinyl thiols on the one hand, and *S*-nitrosoalbumin (SNALB) on the other hand. PTM also occur on many of the Tyr residues to form 3-nitrotyrosine (NTALB). Based on the nM-concentrations of SNALB (< 200 nM; Tsikas et al. 1999a, 2002; Tsikas 2008) and NTALB (< 20 nM) (Tsikas and Duncan 2014) in plasma of healthy and ill subjects, *S*-nitrosylation of Cys^34^ and nitration of Tyr in albumin are considered rather minor from a quantitative point of view. The pathophysiological roles of NTALB remain still unexplored; circulating NTALB is considered a biomarker of oxidative and nitrosative stress (Tsikas and Duncan 2014). In contrast to NTALB, circulating SNALB is considered a major reservoir of nitric oxide (NO)-related bioactivity due to its potential to release NO from its *S*-nitroso group on Cys^34^ under certain conditions possibly leading to vasodilation and platelet anti-aggregation (Giustarini et al. 2007). Authentic SNALB is not a NO-donor on its own. Free l-cysteine was found to mediate the release of NO from SNALB in vitro and the reduction of blood pressure in vivo in the rat (Warnecke et al. 2009). Using ODQ, which is considered a relatively specific inhibitor of the soluble guanylyl cyclase (sGC), SNALB and *S*-nitroso-cysteine (CysSNO) were found to inhibit the aggregation of human platelets in platelet-rich plasma and of washed platelets in part by activating intra-platelet sGC and in part by inhibiting intra-platelet synthesis of thromboxane A_2_ (TxA_2_) (Tsikas et al. 1999a, b, c), one of the most potent endogenous activators of platelet aggregation, an antagonist of NO and prostacyclin (PGI_2_).

l-Cysteine reacts with the *S*-nitroso group of SNALB to form CysSNO via a reversible *S*-transnitrosylation reaction (Tsikas et al. 1999c). CysSNO is specifically transported into cells (Li et al. 2007). CysSNO formed in erythrocytes can also be exported and *S*-transnitrosylate albumin in plasma to form SNALB (Sandmann et al. 2005). A cystine-cysteine shuttle has been reported to facilitate cellular responses to SNALB (Zhu et al. 2008). CysSNO is a very labile *S*-nitrosothiol (RSNO) and may release relatively high amounts of NO, presumably due to mediation of very low amounts of Cu^1+^ ions that can be generated by reduction of Cu^2+^ ions by l-cysteine (CysSH). *S*-Transnitrosylation reactions occur between different endogenous and exogenous *S*-nitrosothiols and thiols (RSH) (Tsikas et al. 1999c). In theory, other endogenous cysteine-based low-molecular-mass (LMM) thiols such as glutathione (GSH), cysteinylglycine (CysGly), homocysteine (hCysSH) and *N*-acetylcysteine (NAC) could also mediate “activation” of SNALB. Such an effect could also be assumed for the synthetic cysteinyl thiols *N*-acetylcysteine ethyl ester (NACET) and cysteine ethyl ester (CysSH-Et). Previously, we showed using a modified aortic ring assay that some of these thiols may modulate the vasodilatory potency of *S*-nitrosothiols (Giustarini et al. 2011). In the present study, we investigated the effects of the above mentioned thiols in comparison to CysSH on the anti-aggregatory effects of synthesized and purified SNALB on platelet aggregation. In these experiments, ODQ was used to investigate a possible involvement of intra-platelet sGC in the anti-platelet effect of SNALB alone and in combination with the tested LMM thiols.

## Materials and methods

### Materials

Sodium [^15^N]nitrite (98% at ^15^N) was purchased from Cambridge Isotope Laboratories (Andover, MA, USA). Sodium [^15^N]nitrate (99% at ^15^N) was supplied from MSD Isotopes Merck Frosst Canada (Montreal, Canada). Glutathione (GSH), l-cysteine, d-cysteine, l-homocysteine, *N*-acetyl-l-cysteine (NAC), dithiothreitol (DTT), sodium nitroprusside (SNP) and hemoglobin (Hb) were purchased from Sigma (Munich, Germany). *N*-Acetyl-l-cysteine ethyl ester (NACET) was prepared as described elsewhere (Giustarini et al. 2012; Tsikas et al. 2018). HiTrapBlue Sepharose affinity columns (1-mL for quantitative analyses and 5-mL cartridges for isolation of human plasma albumin (ALB) and freshly prepared SNALB and S^15^NALB) were obtained from Pharmacia Biotech (Freiburg, Germany). Centrisart I^®^ ultrafiltration cartridges (pore size 4 μm, cut-off 20 kDa) were obtained from Sartorius (Göttingen, Germany). NO gas and other chemicals including sodium nitrite and buffer salts were purchased from Merck (Darmstadt, Germany). ODQ was obtained from ALEXIS Corporation (San Diego, CA, USA). Stock solutions of ODQ were prepared in DMSO. Collagen was obtained from Hormonchemie (Munich, Germany).

### Synthesis of *S*-nitrosothiols

SNALB and S^15^NALB standards were prepared by incubating albumin extracted from freshly obtained human plasma with unlabeled and ^15^N-labelled *n*-butyl nitrite, respectively, and were isolated, characterized and standardized by gas chromatography-mass spectrometry (GC–MS) as described previously (Tsikas et al. 1999a). Typical SNALB concentrations in stock solutions in physiological saline were about 150–170 μM. Solutions (10 mL, 10 mM) of LMM thiols were prepared in physiological saline, aliquoted (0.5 mL) and stored at − 20 °C. CysSNO, GSNO, *S*-nitroso-*N*-acetyl-L-cysteine (NACysSNO), CysS^15^NO, GS^15^NO and NACysSNO (NACysS^15^NO) were freshly prepared by mixing equimolar solutions in physiological saline of l-cysteine, GSH or NAC (0.5 mL, 10 mM) and unlabeled or ^15^N-labelled nitrite (0.5 mL, 10 mM) and by acidifying with hydrochloric acid at a final concentration of 50 mM as described elsewhere (Tsikas et al. 1999b). Solutions of thiols and their *S*-nitrosothiols were stored in an ice-bath in the dark until immediate use. The concentrations in their stock solutions were confirmed by HPLC with UV absorbance detection as described (Tsikas et al. 1999b). Remaining LMM thiols and *S*-nitrosothiols in their stock solutions and dilutions were discarded.

### Measurement of platelet aggregation

Blood platelets were isolated from EDTA-anticoagulated venous blood (10 mL) from healthy volunteers who had not received aspirin or other non-steroidal anti-phlogistic drugs for at least 10 days. Informed written consent was obtained from all volunteers. Platelet aggregation measurements were performed using freshly prepared washed platelet suspensions as described elsewhere (Tsikas et al. 1999d). All assays were carried out within 3 h of their preparation. During the aggregation measurements washed platelets were stored at room temperature under gentle shaking. Briefly, washed platelet suspensions (250 μL aliquots adjusted to contain approximately 4 × 10^8^ cells) were incubated for 3 min in duplicate and constant stirring at 37 °C with 0.9 wt% NaCl (i.e., the control), SNALB, thiols, or ODQ at varying concentrations. When ODQ was used, platelets were pre-incubated for 3 min, before the addition of the tested drugs. Platelet aggregation was induced by collagen (1.0 μg/mL) and was monitored for 5 min by measuring the increase in light transmission with an Apact dual-channel aggregometer (LAbor, Hamburg, Germany) in two cuvettes according to the method of Born and Cross (Born and Cross 1963). The final portion of DMSO, which was used to dissolve ODQ, in the platelet suspension was less than 3 vol% and did not influence platelet aggregation.

### Statistical analysis

Values are expressed and presented as mean ± standard deviation. The significance of differences was determined using Mann–Whitney test. A *P* value of < 0.05 was considered significant.

## Results

CysSH, CysSH-Et, hCysSH, CysGly and GSH each at 10 µM did not change considerably the collagen-induced aggregation of washed human platelets in the absence of SNALB or in the absence of ODQ as compared to physiological saline which served as a control; maximum aggregation: 69 ± 4, 61 ± 5, 68 ± 2, 67 ± 3, 69 ± 4 and 73 ± 6% (each *n* = 2), respectively. CysSH-Et seems to have the strongest effect among the tested thiols albeit weak.

**Fig. 1:**
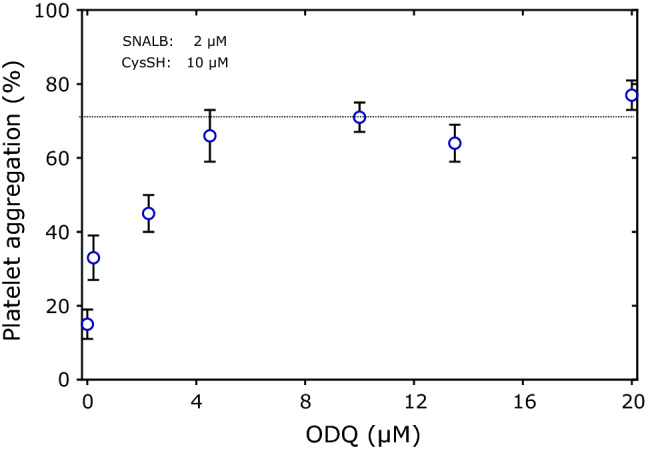
Effect of ODQ on the inhibitory action of SNALB (2 µM) + CysSH (10 µM) on collagen-induced (1.0 µg/mL) aggregation of washed platelets (4 × 10^8^ cells). Data are shown as mean with SD from duplicate measurements in parallel

In the literature, the sGC inhibitor ODQ is commonly used at a concentration of 10 µM in platelet aggregation and vasodilation experiments to demonstrate involvement of NO. We investigated this issue in the present study in the ODQ concentration range of 0–20 µM. The results of Fig. 1 show that ODQ reverses the SNALB + CysSH-induced inhibition of collagen-induced aggregation of washed platelets in a concentration dependent manner. The maximum inhibitory effect of ODQ seems be in the range 4–20 µM. In all subsequent investigations of the present study, ODQ was used at the fixed concentration of 10 µM, which is considered to be sufficient and also to minimize potential effects of the solvent DMSO.

**Fig. 2:**
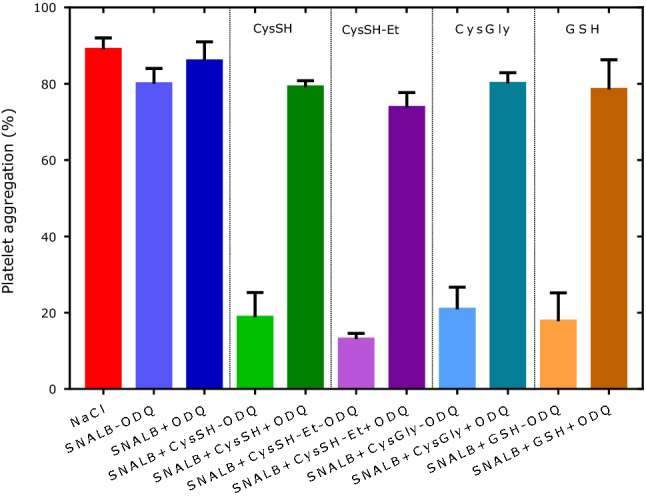
Effect of SNALB (2 µM) and thiols (10 µM) on collagen-induced (1.0 µg/mL) washed platelets (4 × 10^8^ cells) aggregation in the absence or in the presence of ODQ (10 µM). Physiological saline was used as a control (NaCl, maximum aggregation). Thiols were dissolved in physiological saline, and ODQ in DMSO. Data are shown as mean with SD from duplicate measurements (parallel use of two cuvettes)

ALB (ALB-CysSH, at 2 µM) alone and in combination with the individual LMM thiols (each at 10 µM) had no effect on the collagen-induced (1 µg/mL) aggregation (data not shown) and was not further used in the study. The effects of CysSH, CysSH-Et, CysGly and GSH (each at 10 µM) on SNALB (at 2 µM) were investigated in the absence or in the presence of ODQ (at 10 µM). The results of this experiment are shown in Fig. 2. SNALB had a weak inhibitory effect on platelet aggregation in the absence of ODQ. All LMM thiols reduced maximum aggregation, with CysSH-Et apparently being a little stronger enhancer than the other tested thiols. In the presence of ODQ, the effects of all thiols were almost entirely reversed.

DMSO may inhibit platelet aggregation (White et al. 1974; Saeed et al. 1988). As DMSO was used to dissolve ODQ, we tested potential effects of DMSO. In the absence of ODQ but in the presence of DMSO (0.5, 1, 2, 3, 4 vol%), the extent of aggregation of SNALB (2 µM) + CysSH (10 µM) was determined to be 18 ± 4.5%. In the presence of ODQ (2.25, 4.5, 9, 13.5, 20 µM) added in DMSO in volumes resulting in final DMSO concentrations of 1, 2, 3, 4 vol%, respectively, the extent of aggregation of SNALB (2 µM) + CysSH (10 µM) was determined to be 64 ± 12%. There was no correlation between platelet aggregation and DMSO portion (*r* = 0.3, *P* = 0.68) or ODQ concentration (*r* = 0.7, *P* = 0.23). These results suggest that DMSO has no appreciable effects on collagen-induced aggregation under the conditions used in our study at volume contents up to 3%.

**Fig. 3:**
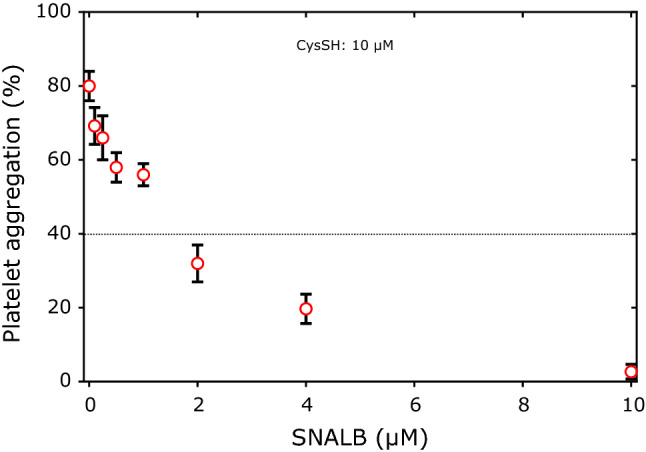
Effect of SNALB (range, 0–10 µM) and CysSH (10 µM) on collagen-induced (1.0 µg/mL) aggregation of washed platelets (4 × 10^8^ cells). Data are shown as mean with standard deviation from duplicate parallel measurements

Previously, we found that CysSH induces release of NO from SNALB in potassium phosphate buffer of pH 7.4 in a manner depending on the concentration of CysSH (range, 0–500 µM) and SNALB (range, 0–20 µM) with an approximate yield of 15% with respect to SNALB (Warnecke et al. 2009). In the resent study, we investigated this issue by measuring collagen-induced aggregation of washed platelets. At the fixed CysSH concentration of 10 µM platelet aggregation decreased with increasing initial concentration of SNALB added to the washed platelet suspension (Fig. 3).

Representative tracings obtained from platelet aggregation measurements on human washed platelets using SNALB and CysSH are shown in Fig. S1 (Supplement).

**Fig. 4:**
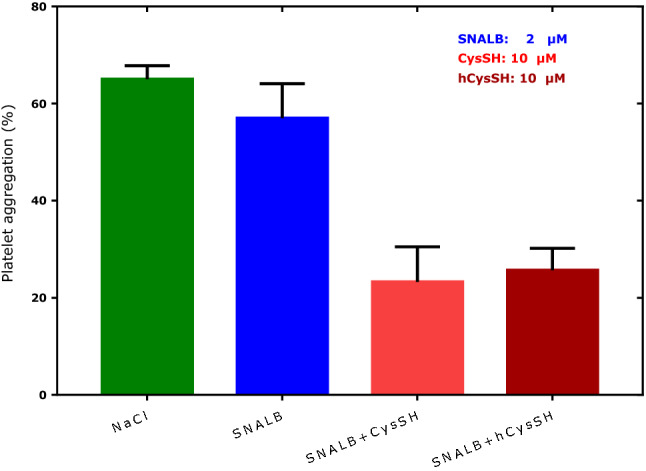
Effect of SNALB (2 µM), CysSH (10 µM) and hCysSH (10 µM) on collagen-induced (1.0 µg/mL) aggregation of washed platelets (4 × 10^8^ cells). Physiological saline was used as a control (maximum aggregation). Data are shown as mean with standard deviation from quadruplicate parallel measurements

hCysSH is an endogenous thiol which occurs in plasma of healthy humans in its free form at concentrations below 1 µM, which is about 20 times lower than that of free plasma CysSH (Giustarini et al. 2012). We compared in parallel the effects of CysSH and hCysSH on the inhibition of collagen-induced platelet aggregation by SNALB. Figure 4 shows that CysSH and hCysSH are equally potent effectors of SNALB-related inhibition of collagen-induced aggregation of human washed platelets.

**Fig. 5:**
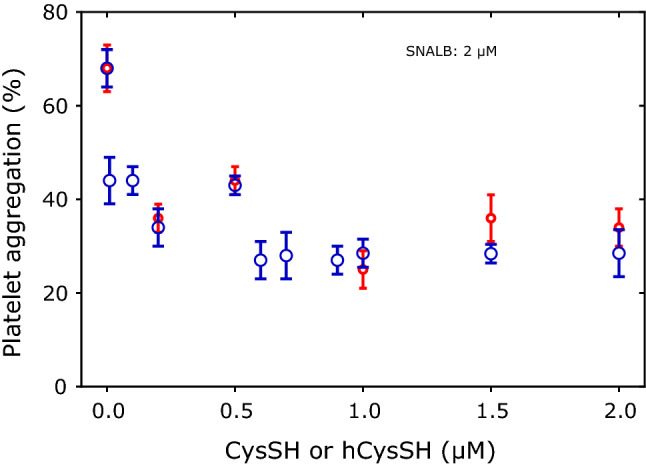
Effect of SNALB (2 µM), CysSH (range, 0—2 µM; blue circles) and hCysSH (range, 0–2 µM; red circles) on collagen-induced (1.0 µg/mL) aggregation of washed platelets (4 × 10^8^ cells). Data are shown as mean with standard deviation from duplicate parallel measurements

We investigated in parallel the effects of CysSH and hCysSH on the SNALB-dependent inhibition of collagen-induced aggregation in washed human platelets in the narrow range of 0–2 µM. Figure 5 shows a similar activity profile of CysSH and hCysSH.

**Fig. 6:**
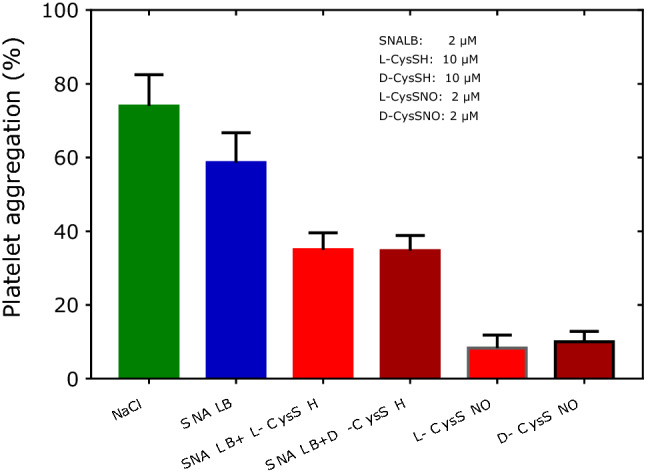
Effect of SNALB alone (2 µM) and in combination with l-CysSH (10 µM), d-CysSH (10 µM), and of l-CysSNO (2 µM) and D-CysSNO (2 µM) on collagen-induced (1.0 µg/mL) aggregation of washed platelets (4 × 10^8^ cells). Data are shown as mean with standard deviation (*n* = 2–6)

To investigate potential effects of the stereochemistry of thiols on the SNALB-related inhibition of platelet aggregation, we used the L- and D-forms of CysSH, i.e., l-CysSH and d-CysSH, as well as their *S*-nitrosothiols, i.e., l-CysSNO and d-CysSNO. Figure 6 shows that l-CysSH and d-CysSH were equally potent enhancers of the anti-aggregatory potential of SNALB (*P* = 0.974, Mann–Whitney test). Also, the corresponding *S*-nitrosothiols, l-CysSNO and d-CysSNO, were equally potent inhibitors of collagen-induced aggregation and several times more potent than the combination of SNALB with l-CysSH or d-CysSH.

**Fig. 7:**
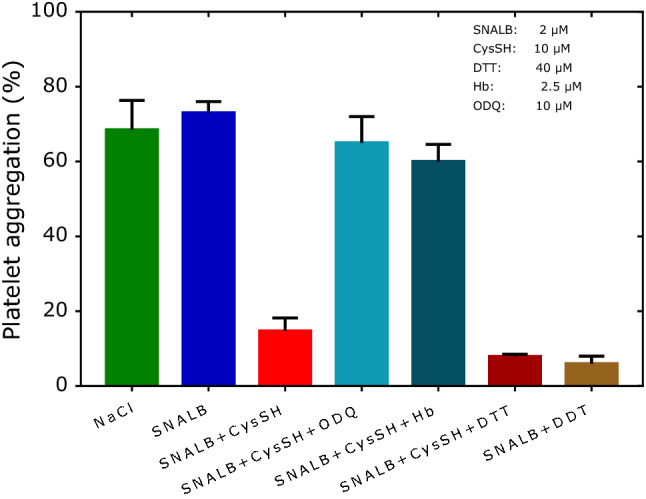
Effect of SNALB alone (2 µM) and in combination with CysSH (10 µM), DTT (40 µM), Hb (2.5 µM) or ODQ (10 µM) on collagen-induced (1.0 µg/mL) aggregation of washed platelets (4 × 10^8^ cells). Data are shown as mean with standard deviation from duplicate measurements

In addition to the above mentioned thiols we also tested the effects of the LMM thiol dithiothreitol (DTT) which contains two sulfhydryl groups and the HMM thiol hemoglobin (Hb). Figure 7 shows that Hb (at 2.5 µM) is as efficient as ODQ (at 10 µM) in ameliorating the effect of CysSH (at 10 µM) on the SNALB-related inhibition of collagen-induced aggregation of washed human platelets. In contrast, DTT seems to slightly enhance the effect of CysSH on SNALB with respect to platelet aggregation.

**Fig. 8:**
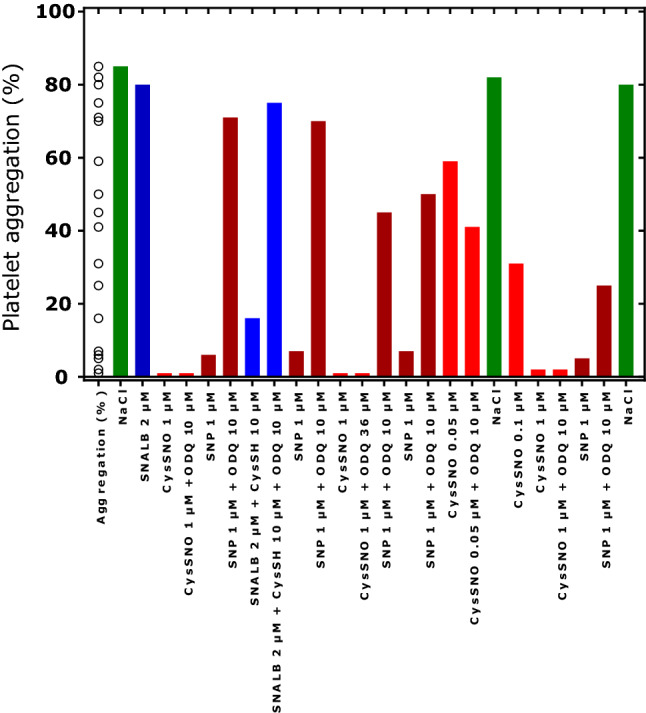
Effect of SNALB (2 µM), CysSNO (0.05, 0.1, 1.0 µM), SNP (1 µM), CysSH (10 µM), ODQ (10 µM; 36 µM) on collagen-induced (1.0 µg/mL) aggregation of washed platelets (4 × 10^8^ cells). Platelets were obtained from a healthy volunteer and used within a single experiment without delay. The substances were used in the indicated order (from the left to the right). ODQ was added to the washed platelets prior to the addition of the test substances and incubated for 5 min. Collagen was added 3 min after the addition of the test substances and platelet aggregation was measured for 5 min. During the measurements the washed platelet suspension was kept at room temperature under gentle shaking. Each column represents a single measurement. The symbols in “Aggregation (%)” on the left indicate the individual values of platelet aggregation measurements

Using a single washed platelet preparation from a healthy human subject, we consecutively tested the effects of SNALB and other substances to test the repeatability of the results within a single experimental setting. The results of this experiment are shown in Fig. 8. Maximum aggregation (NaCl) varied by 3% (82 ± 2.5%, *n* = 3), SNP-induced inhibition (6.3 ± 1.0%, *n* = 4) varied by 15%, the combination SNP + ODQ (53 ± 22%, *n* = 4) varied by 42%, CysSNO-induced inhibition without ODQ (1.3 ± 0.6%, *n* = 4) varied by 43%, and with ODQ (1.5 ± 0.7%, *n* = 2) by 47%. SNP (at 1 µM) inhibited potently collagen-induced platelet aggregation; ODQ (at 10 µM) partly reversed SNP’s effect (*P* = 0.029, Mann–Whitney test). Apparently, the antagonistic effect of ODQ on SNP attenuated in this experiment during the measurements (71%, 70%, 45%, 25%). CysSNO inhibited collagen-induced platelet aggregation in a concentration-dependent manner and its action was not reversed by ODQ (at 10 and 36 µM). This finding confirms the results of a previous study (Tsikas et al. 1999d). Interestingly, the inhibitory action of the combination of SNALB (2 µM) and CysSH (10 µM) was reversed by ODQ (10 µM) (Fig. 8), suggesting involvement of at least an alternative mechanism.

## Discussion

The mechanisms of the formation and biological activity of SNALB have been widely investigated over the last almost 30 years, but they are not entirely understood. As the SH group of ALB cannot react with NO, it is likely that SNALB is formed by the reaction of nitrous anhydride (N_2_O_3_) (R1). N_2_O_3_ is extremely labile in aqueous media and is considered to be an intermediate of the autoxidation of NO (R2). N_2_O_3_ is extremely reactive against thiols and amines, i.e., a potent *S*- and *N*-nitrosating species. The reaction of N_2_O_3_ with the Cys^34^SH group of ALB would lead to the formation of SNALB among other species (R1). SNALB can also be formed by reversible *S*-transnitrosylation reactions of ALB with endogenous LMM *S*-nitrosothiols such as *S*-nitrosocysteine (CysSNO) and *S*-nitrosoglutathione (GSNO) (R3). LMM *S*-nitrosothiols can be formed from the *S*-nitrosylation of endogenous thiols and N_2_O_3_ according to reaction (R1). Recently, GSNO and CysSNO were shown to be formed from nitrite (NO_2_^−^) in buffered carbonic anhydrase (CA) solutions in the presence of GSH or CysSH (Hanff et al. 2016, 2018; Zinke et al. 2016). Thus, *S*-nitrosylation (R1) and *S*-transnitrosylation (R3) of CysSH moieties of proteins could be considered as PTM. We have previously shown that SNALB in plasma can be formed by transmembraneous *S*-transnitrosylation of plasma albumin by *S*-nitrosothiols formed in human red blood cells (Sandmann et al. 2005).

1 $${\text{ALB}} - {\text{Cys}}^{{34}} {\text{SH }} + \, \left[ {{\text{N}}_{2} {\text{O}}_{3} } \right] \to {\text{ALB}} - {\text{Cys}}^{{34}} {\text{SNO }} + {\text{ NO}}_{2}^- + {\text{ H}}^+$$

2 $${\text{4 NO }} + {\text{ 2 H}}_{2} {\text{O}} \to {2 }\left[ {{\text{N}}_{2} {\text{O}}_{3} } \right] \to {\text{4 NO}}_{2}^- + {\text{ 4 H}}^+$$

3 $${\text{ALB}} - {\text{Cys}}^{{34}} - {\text{SH }} + {\text{ GSNO}} \to \leftarrow {\text{ALB}} - {\text{Cys}}^{{34}} - {\text{SNO }} + {\text{ GSH}}$$

4 $${\text{ALB}} - {\text{Cys}}^{{34}} {\text{S}} - {\text{NO }} + {\text{ Cu}}^{{1} + } \to {\text{ALB}} - {\text{Cys}}^{{34}} {\text{S}} - {\text{SH }} + {\text{ NO }} + {\text{ Cu}}^{{2} + }$$

SNALB is not an NO donor on its own (Tsikas et al. 2002; Warnecke et al. 2009) and can, therefore, not exert biological activity through NO release. Yet, SNALB was found in vitro to inhibit platelet aggregation (Gordge et al. 1996; Tsikas et al. 1999b) and to lower blood pressure in vivo in the rat (Warnecke et al. 2009). These observations suggest that SNALB may exert biological activity at least through two mechanisms: (1) by *S*-transnitrosylating SH groups on the surface of cells, and (2) by releasing NO after reduction of its *S*-nitrosyl group (NO^+^). The latter mechanism has been suggested to explain the anti-platelet function of GSNO and SNALB and to require the catalytic action of a Cu^1+^-dependent enzyme (Gordge et al. 1996; Gordge & Xiao 2010) (R4). The putative platelet enzyme has not been identified thus far.

The use of ODQ, an inhibitor of sGC, and the measurement of platelet cGMP, the reaction product of sGC, and the measurement of NO revealed that *S*-nitrosothiols including CysSNO and SNALB may inhibit platelet aggregation by cGMP-dependent (via NO) and cGMP-independent mechanisms presumably including *S*-transnitrosylation reactions and inhibition of platelet cyclooxygenase-catalyzed synthesis of thromboxane A_2_, an endogenous potent inductor of platelet aggregation (Tsikas et al. 1999d). Given the strong potency of CysSNO to release NO and to inhibit human platelet aggregation (Tsikas et al. 1999d), we have hypothesized that the LMM CysSNO may be the active principle of the bioactivity of SNALB. The *S*-transnitrosylation of CysSH by SNALB would lead to formation of CysSNO (R5) which is known to “spontaneously” decompose to NO. Because the latter reaction is dependent on CysSH and Cu^2+^, we further hypothesized that only very small amounts of the strong reductor Cu^1+^ would be required for this reaction (R6), analogous to the Cu^1+^-dependent yet still unknown enzyme (Gordge et al. 1996; Gordge and Xiao 2010). Cu^1+^ can be easily provided through the reaction of CysSH with free Cu^2+^ (R7) and protein-associated Cu^2+^ such as in ceruloplasmin (Feldman et al. 1982). The present study (Fig. 8) and a previous study from our group (Tsikas et al. 1999d) suggest that CysSNO and the combination of SNALB and CysSH differ in their anti-aggregatory action with respect to ODQ, the inhibitor of sGC. This significant difference suggests that the mechanisms by which CysSNO and SNALB + CysSH inhibit collagen-induced aggregation of washed human platelets are different. It is possible that the different effects of ODQ are associated with different concentrations of active species including authentic CysSNO, CysSNO formed from SNALB + CysSH, and NO. It was reported that extracellular NO concentrations of more than 40 nM are required for cGMP-independent inhibition of activation of washed human platelets (Crane et al. 2005). In our study this is expected for CysSNO rather than for SNALB + CysSH.

5 $${\text{ALB}} - {\text{Cys}}^{{34}} - {\text{SNO }} + {\text{ CysSH}} \leftarrow \to {\text{ALB}} - {\text{Cys}}^{{34}} - {\text{SH }} + {\text{ CysSNO}}$$

6 $${\text{CysSNO }} + {\text{ Cu}}^{{1} + } \to {\text{CysSH }} + {\text{ NO }} + {\text{ Cu}}^{{2} + }$$

7 $${\text{2 CysSH }} + {\text{ 2 Cu}}^{{2} + } \leftarrow \to \left( {{\text{CysS}}} \right)_{2} + {\text{ 2 Cu}}^{{1} + } + {\text{ 2 H}}^+$$

Unlike CysSNO, the *S*-nitrosothiol of its homolog homocysteine, i.e., hCysSNO, GSNO and the drug sodium nitroprusside (SNP) are much poorer NO donors on themselves (Sandmann et al. 2005). Yet, they are potent inhibitors of platelet aggregation and vasodilators. In the present study we investigated the effects of various endogenous and exogenous cysteinyl thiols on the anti-platelet activity of SNALB. To minimize effects of other components such as ceruloplasmin, a Cu^2+^-rich plasma protein (Inoue et al. 1999; Crane et al. 2005), free Cu^2+^ ions (Stubauer et al. 1999), and to maximize the anti-platelet effects of SNALB and other *S*-nitrosothiols by lowering the protein binding (Giustarini et al. 2012), we performed aggregation measurements using washed platelets instead of platelet-rich plasma. It is known that albumin binds on the surface of platelets (Kelton and Steeves 1983). Presumably, SNALB also binds to platelet surface components and may compete with albumin, but also to interact with additional groups such as free SH groups via its *S*-nitroso group. In our study, ODQ did not inhibit the anti-aggregatory action of authentic NO when added to human washed platelets at concentrations above 1 µM (data not shown). High extracellular NO concentrations are likely to inhibit platelet activation via cGMP-independent mechanisms.

Using SH- and SS-specific agents such as *N*-ethylmaleimide (NEM) and dithiothreitol (DTT), respectively, it was observed that protein sulfhydryl (PSH) and disulfide (PSSP) groups of the platelet surface are involved in aggregation processes via thiol exchange reactions (Margaritis et al. 2011). NEM was found to inhibit aggregation of washed platelets by about 20% (collagen as inductor) and about 90% (ADP as inductor) at 20 µM and to almost 100% at 50 µM, suggesting that covalent alkylation of SH groups by NEM inhibits platelet aggregation (Margaritis et al. 2011). NEM was found to be several times more potent in washed platelets compared to platelet-rich plasma, most likely due the higher molar ratio of NEM-to-SH groups in washed platelets. One could suggest that the *S*-transnitrosylation of SH groups on the surface of platelets by *S*-nitrosothiols resembles the antiplatelet effect of NEM although *S*-transnitrosylation is reversible. DTT itself was found to induce platelet aggregation, albeit at mM-concentrations, presumably by reducing PSSP to PSH. In our study we used DTT at much lower µM-concentrations (40 µM). At this concentration DTT increased the antiplatelet effect of SNALB presumably via *S*-transnitrosylation of platelet SH groups by *S*-nitroso-DTT formed by the reaction of SNALB with DTT (R8). Because of the higher DTT concentration compared to CysSH (40 vs 10 µM), it is possible that DTT-SNO is the predominant species in the mixture of SNALB (2 µM), CysSH (10 µM) and DTT (40 µM) used in our study. Identified platelet surface proteins that are necessary for platelet aggregation were found to include glycoprotein VI (GPVI; collagen receptor), P_2_Y_12_ (ADP receptor) and integrin αIIbβ3 (Margaritis et al. 2011). It is possible that these proteins have also been targeted by the *S*-nitrosothiols in our present and previous studies from our group (Tsikas et al. 1999d).

8 $${\text{ALB}} - {\text{Cys}}^{{34}} - {\text{SNO }} + {\text{ DTT}} \leftarrow \to {\text{ALB}} - {\text{Cys}}^{{34}} - {\text{SH }} + {\text{ DTT}} - {\text{SNO}}$$

In our experiments, l-CysSNO and d-CysSNO were equally effective as inhibitors of washed platelet aggregation. l-CysSH and d-CysSH were also equally effective in enhancing the anti-platelet action of SNALB. Previously, we found that l-CysSNO and d-CysSNO are comparably strong NO donors and to yield comparable nitrite concentrations in platelet-rich plasma (Tsikas et al. 1999d). These observations together suggest that l-CysSNO and d-CysSNO are not exclusively transported into the washed platelets, but their inhibitory actions also take place on the surface of the platelets. Unlike CysSNO, GSNO is not transported into erythrocytes (Sandmann et al. 2005). Whether this also applies to platelets is not known. GSNO itself is a poor NO donor. It is likely that GSNO inhibits platelet aggregation by *S*-nitrosylating SH groups on the surface of platelets like other *S*-nitrosothiols including CysSNO and hCysSNO. SNP itself is also a poor NO donor. SNP was found to inhibit collagen-induced aggregation of washed platelets in a cGMP-dependent and cGMP-independent manner (Tsikas et al. 1999d) presumably including inhibition of TxA_2_ synthesis as reported by others (Levin et al. 1982). In our present study, SNP (1 µM) was found to potently inhibit collagen-induced aggregation mainly in a cGMP-dependent manner. To our knowledge it is not known whether SNP is transported across cell membranes. The anti-platelet action of SNP could involve reaction with SH groups of proteins on the platelet surface which is likely to oxidize rather than to nitrosylate it as demonstrated by us for albumin (Tsikas et al. 2001).

**Scheme 1:**
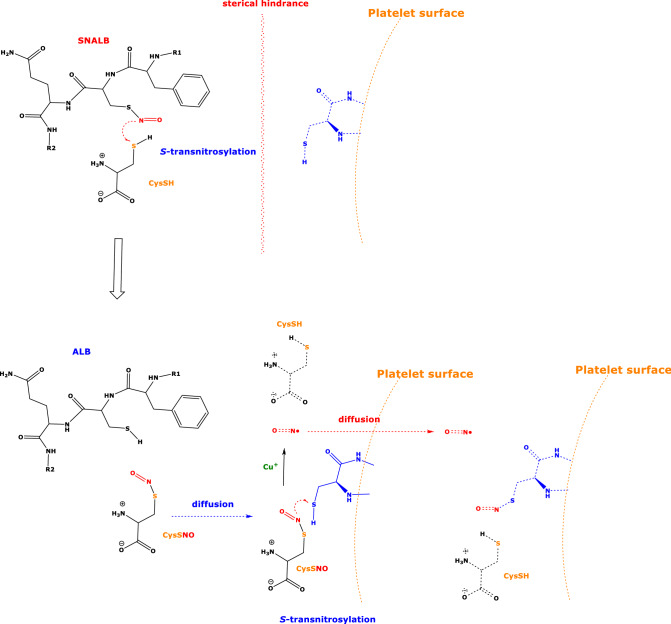
Simplified illustration of the cysteine-mediated *S*-transnitrosylation of the platelet surface by *S*-nitrosoalbumin (ALB-Cys^34^SNO, SNALB). The interaction of the *S*-nitroso group of ALB-CysSNO with SH groups of cysteine moieties of the platelet surface is sterically hindered. The SH group of cysteine (CysSH) is *S*-transnitrosylated by ALB-Cys^34^SNO to form albumin (ALB) and *S*-nitroso-cysteine (CysSNO). CysSNO diffuses to the platelet surface and *S*-transnitrosylates a cysteine moiety of the platelet surface. CysSNO is also supposed to be converted by Cu^+^ to nitric oxide (NO) and CysSH. *S*-Transnitrosylation of SH groups of the platelet surface and NO formed outside the platelet inhibit collagen-induced platelet aggregation by several intra-cellular mechanisms (not shown)

The effect of SNALB (at 2 µM) on collagen-induced washed platelet aggregation observed in the present work and in a previous study from our group (Warnecke et al. 2009) lets SNALB appear as a very weak cGMP-dependent inhibitor of aggregation of human platelets (Scheme 1). Possible explanations for this could be very low-extent (1) release of NO from SNALB, and (2) *S*-transnitrosylation of SH groups on the platelet surface by SNALB. Very low extent of NO release from SNALB prepared and purified in our laboratory could be due to impurities by LMM thiols in the washed platelet preparation, perhaps in synergism with Cu^2+^ firmly bound to SNALB that was not removed by the main purification step, that is, the ALB-specific extraction of SNALB on HiTrapBlue Sepharose cartridges (Tsikas et al. 1999b). The Cys^34^ group of albumin is located in its N terminal of loop I. It can, therefore, be a considerable steric hindrance for the *S*-nitroso group of the bulk ALB-Cys^34^SNO to approach SH groups on the surface of the platelet the size of which is about 140–200 times larger (platelet largest diameter: 2000–3000 nm) than HSA (length: 14 nm; Carter et al. 1989). In contrast, all LMM *S*-nitrosothiols investigated in the present study are likely to readily approach SH groups located on the surface of the platelets. Protein disulfide isomerase (PDI, 57 kDa) has four active cysteine residues (Cys^53^, Cys^56^, Cys^397^, Cys^400^) of which Cys^397^ and Cys^400^ are *S*-nitrosylated by GSNO to form *S*-nitroso protein disulfide isomerase (PDI-CysSNO) (Bekendam et al. 2018). PDI-CysSNO was shown to inhibit (at 250 nM) aggregation of washed platelets induced by the protease-activated receptor-1-activating peptide SFLLRN (Bekendam et al. 2018), suggesting that proteins of size comparable to that of ALB may have different access to SH groups on the platelet surface.

CysSH, GSH and Cu^2+^ are of particular importance in the Wilson’s disease; treatment with the synthetic thiol penicillamine (chelator) and exogenous Zn^2+^ (substitution of Cu^2+^ from its stores) have favorable effects on this disease (Farinati et al. 2003). CysSH, GSH, Zn^2+^ and Cu^2+^ also play a key role in the nitrous anhydrase activity of carbonic anhydrase which is associated with formation of N_2_O_3_, GSNO, CysSNO and NO (Hanff et al. 2016, 2018; Zinke et al. 2016; Tsikas 2021; Tsikas and Gambaryan 2021). Intra-platelet carbonic anhydrase seems to be of significance in the pharmacological nitrite-dependent inhibition of platelet aggregation via *S*-transnitrosylation reactions (Tsikas and Gambaryan 2021). Yet, involvement and importance of carbonic anhydrase and its PTM including *S*-transnitrosylation in the Wilson’s disease have been sparingly investigated thus far (Di Fiore et al 2020).

## Conclusions

Nitrous anhydride (N_2_O_3_) is an intermediate in the autoxidation of nitric oxide (NO) to nitrite and of the nitrous anhydrase activity of carbonic anhydrase. It is a potent *S*-nitrosating species that generates *S*-nitrosoproteins and LMM *S*-nitrosothiols. The most characteristic feature of *S*-nitrosothiols is the non-enzymatic *S*-transnitrosylation of proteins. Based on the pM-to-nM concentrations of *S*-nitrosothiols in blood and tissue, *S*-transnitrosylation of proteins could be considered as a negligible post-translational modification (PTM). Yet, these chemical non-redox reactions are very rapid, reversible, and involved in cell-signaling, often compared with enzymatic phosphorylation reactions. In human blood, *S*-nitrosohemoglobin and *S*-nitrosoalbumin (SNALB) are the most abundant *S*-nitrosoproteins. They are assumed to store and transport NO-related bioactivity through the body. SNALB itself can inhibit collagen-induced aggregation of human washed platelets to a minor extent. LMM cysteinyl thiols such as CysSH and GSH potentiate the anti-aggregatory action of SNALB at concentrations commonly found in human plasma. The underlying mechanism involves *S*-transnitrosylation of LMM cysteinyl thiols by SNALB to form the corresponding LMM *S*-nitrosothiols which because of their very small size, can freely *S*-transnitrosylate proteinic SH groups on the platelet surface. This signals to the platelet to start several cascades including activation of the sGC to form cGMP, inhibition of TxA_2_ synthesis, and inhibition of the TxA_2_ receptor to eventually inhibit platelet aggregation.

## Supplementary Information

Below is the link to the electronic supplementary material.

Supplementary file1 (DOCX 276 KB)

## Abbreviations

ADP: Adenosine diphosphate
ALB: Albumin
CysSH: L-Cysteine or d-cysteine
CysSH-Et: L-Cysteine ethyl ester
CysGly: Cysteinylglycine
hCysSH: L-Homocysteine
DTT: Dithiothreitol
GC–MS: Gas chromatography-mass spectrometry
cGMP: Cyclic guanosinemonophosphate
GSH: Glutathione
GSNO: *S*-Nitrosoglutathione
Hb: Hemoglobin
HMM: High-molecular-mass
HSA: Human serum albumin
LMM: Low-molecular-mass
*m*/*z*: Mass-to-charge ratio
NAC: *N*-Acetyl-L-cysteine
NACET: *N*-Acetyl-L-cysteine ethyl ester
NEM: *N*-Ethylmaleimide
NO: Nitric oxide
NTALB: 3-Nitroalbumin
ODQ: 1*H*-[1,2,4]Oxadiazolo[4,3-*a*]quimoxalin-1-one
PDI: Protein disulfide isomerase
PFB: Pentafluorobenzyl
RSH: Thiol
RSNO: *S*-Nitrosothiols
SH: Sulfhydryl
sGC: Soluble guanylyl cyclase
SIM: Selected-ion monitoring
SNALB: *S*-Nitrosoalbumin
CysSNO: *S*-Nitrosocysteine
