# Supplementary material for: Extra-platelet low-molecular-mass thiols mediate the inhibitory action of S-nitrosoalbumin on human platelet aggregation via S-transnitrosylation of the platelet surface
Source: Amino Acids. 2021 Feb 14;53(4):563–73. doi: 10.1007/s00726-021-02950-8 (PMC8107154; doi:10.1007/s00726-021-02950-8)
Supplement: Supplementary file 1 — Supplementary file1 (DOCX 276 KB) [file 726_2021_2950_MOESM1_ESM.docx]

**Supplement to**

**Extra-platelet low-molecular-mass thiols mediate the inhibitory action of *S*-nitrosoalbumin on human platelet aggregation via *S*-transnitrosylation of the platelet surface**

[Dimitrios Tsikas](https://www.sciencedirect.com/science/article/pii/S1570023208008702?via%3Dihub#!)^*^

Institute of Toxicology, Core Unit Proteomics, Hannover Medical School, Carl-Neuberg-Str. 1, 30625 Hannover, Germany

Figure S1. Representative tracings obtained from platelet aggregation measurements on human washed platelets using *S*-[^15^N]nitroso-Cys^34^-albumin (S^15^NALB) alone and in combination with L-cysteine (CysSH) at the indicated concentrations. Physiological saline served as a control to observe the maximal aggregation. Aggregation of washed platelets (about 4×10^8^ cells per measurement) prepared from blood of a healthy volunteer was induced by collagen (1.0 µg/mL). Inserted numbers indicate the run number of the platelet aggregation measurements which were performed on a single setting using a single preparation.
